# Blue Marble Health: A Call for Papers

**DOI:** 10.1371/journal.pmed.1001682

**Published:** 2014-07-29

**Authors:** Peter J. Hotez, Larry Peiperl

**Affiliations:** 1 Sabin Vaccine Institute and Texas Children's Hospital Center for Vaccine Development, Departments of Pediatrics and Molecular Virology and Microbiology, National School of Tropical Medicine at Baylor College of Medicine, Houston, Texas, United States of America; 2 Departments of Medical Humanities and Biology, Baylor University, Waco, Texas, United States of America; 3 James A. Baker III Institute for Public Policy, Rice University, Houston, Texas, United States of America; 4 Public Library of Science, San Francisco, California, United States of America

## Abstract

In this month's editorial, Peter J. Hotez, Co-Editor-in-Chief of *PLOS Neglected Tropical Diseases* and Larry Peiperl, Chief Editor of *PLOS Medicine*, call for papers on “Blue Marble Health”: research on health problems that affect poorer people irrespective of the overall economic strength of the countries in which they live.

*Please see later in the article for the Editors' Summary*

In May 2014, *PLOS Medicine* joined *PLOS Neglected Tropical Diseases* in launching the Blue Marble Health Collection [1], to which we now invite ongoing manuscript submissions.

The collection (available at http://www.ploscollections.org/bluemarblehealth/) emerged from themes shared by both journals, and discussed in Editorials during 2013. First, a Viewpoint in *PLOS Neglected Tropical Diseases* [2] found that while some neglected tropical diseases (NTDs) such as river blindness, loiasis, African sleeping sickness, and schistosomiasis are largely or exclusively diseases of sub-Saharan Africa, paradoxically, many of the world's highest concentration of NTDs occur in the 20 wealthiest economies—the group of 20 (G20) countries—especially in the mostly hidden pockets of extreme poverty that can be found in the big middle-income nations, such as Indonesia, or in areas of the BRIC countries (Brazil, Russia, India, and China), including northeastern Brazil, northern India, and southwestern China. Moreover, the disease burden from NTDs is alarmingly high in the southern United States (especially in Texas and the Gulf Coast), in areas of Australia with large Aboriginal populations such as the Northern Territory, and in Eastern Europe.

A parallel Editorial in *PLOS Medicine* [3] noted that relative poverty within a society is a stronger predictor of health than aggregate measures of economic power such as gross national product or per capita income. For example, tens of millions of Americans living in poverty, including many people of color, “experience levels of health that are typical of middle-income or low-income countries.” The Editorial concluded that, for many issues that affect the health of people of lower socioeconomic status, clear-cut distinctions between “domestic” and “cross-border” research are becoming increasingly difficult to draw.

Blue Marble Health highlights a shift in current thinking about global health. Increasingly, an approach that emphasizes stark contrasts between “developing” and “developed” countries may no longer reflect reality. Instead, major health disparities—including NTDs, but possibly also other infections such as tuberculosis, as well as noncommunicable diseases—disproportionately affect poor people living among wealthier people. Having evolved from paradigms of international health (in which high-income countries assist lower-income countries) through global health (in which countries work together to address health issues that affect the global community), Blue Marble Health reflects the insight that wherever socioeconomic inequality is pervasive, neglected diseases and other conditions naively assumed to arise only in the context of national poverty will spread. To address these problems, Blue Marble Health calls for attention to socioeconomic as well as biomedical factors—whether in Nigeria, South Africa, Brazil, and India, or in the US, Europe, and Australia.

At its launch in May 2014, the collection included more than 20 Editorials, Viewpoints, Policy Forums, and Research Articles from *PLOS Neglected Tropical Diseases* and *PLOS Medicine*, as well as an Essay from *PLOS Biology*. Each highlights a health disparity that disproportionately strikes poor people living in G20 countries or other countries generally thought of as well-off. The launching of the collection also coincided with the publication of a new *PLOS Neglected Tropical Diseases* Editorial that identifies ten global “hotspots” for endemic and epidemic NTDs [4].

Mahatma Gandhi once stated: “It is a trite saying that one half of the world knows not how the other lives. Who can say what sores might be healed, what hurts solved, were the doings of each half of the world's inhabitants understood and appreciated by the other?” [5] A century later, although the importance of mutual appreciation among diverse populations has only increased, the world no longer divides itself easily into halves on the basis of national boundaries or economic cut-points. Amartya Sen, who was awarded the Sveriges Riksbank Prize in Economic Sciences in 1998, has articulated a distinction between “the difficult problem of assessing the richness of human lives, including the freedoms that human beings have reason to value, and…the much easier exercise of keeping track of incomes and other external resources that persons—or nations—happen to have” [6]. In Sen's words, “human well-being and freedom, and their connection with fairness and justice in the world, cannot be reduced simply to the measurement of GDP and its growth rate, as many people are tempted to do.” The term “Blue Marble Health” reflects an understanding that, as a fundamental prerequisite for well-being and richness in human lives, health must not be limited by simplistically defined boundaries, be they geographical or economic.

Moving forward, we encourage the community of biomedical and social scientists, health economists, health-care professionals, and public health workers to submit papers to us at PLOS that highlight health disparities among low-income and otherwise disadvantaged populations, those who are often forgotten in the world's middle- and high-income countries.

## Abbreviations

G20: group of 20
NTD: neglected tropical disease
